# TProtective Factors for Sustaining Robustness in Older Adults: An 8-Year Multinational Longitudinal Study Using SHARE Data

**DOI:** 10.21203/rs.3.rs-9345466/v1

**Published:** 2026-05-18

**Authors:** Khalil Iktilat, Debbie Rand, Netta Bentur, Aviad Tur-Sinai

**Affiliations:** Tel Aviv University; Tel Aviv University; Tel Aviv University; University of Haifa

**Keywords:** Robustness, ageing, SHARE, sustained robustness, healthy ageing, multidimensional resilience, longitudinal ageing trajectories

## Abstract

**Background:**

Frailty is a widely recognized clinical syndrome of ageing. While most studies emphasize risk factors for frailty, little is known about the protective mechanisms that sustain robustness in later life. This study addresses this gap by identifying longitudinal predictors of robustness among older adults.

**Methods:**

We analyzed eight years of longitudinal data from the Survey of Health, Ageing and Retirement in Europe (SHARE; Waves 4–8, 2011–2020) across 27 countries, including 7,865 adults aged 65–84, stratified into younger-old (65–74) and older-old (75–84) groups. Logistic regression models estimated odds ratios for maintaining robustness, incorporating sociodemographic, health, lifestyle, functional factors.

**Results:**

Robustness declined steadily over time, with 52% of younger-old adults and 26% of older-old adults remaining robust after eight years. Across all models, better self-rated health (OR ≈ 0.72, p < 0.001), fewer chronic conditions (OR ≈ 0.89, p < 0.001), and greater financial security (OR ≈ 1.11, p = 0.018) were the strongest and most consistent predictors of sustained robustness. Engagement in moderate physical activity and participation in social and cognitive activities further contributed to resilience, though their effects were stronger among younger-old adults compared with the older-old.

**Conclusions:**

This study contributes to ageing research by examining sustained robustness, rather than frailty progression alone, as a longitudinal outcome in a large multinational cohort. By highlighting behavioral, psychosocial, and functional determinants of resilience, it frames robustness as a measurable and actionable outcome in ageing research and practice. These findings promote a more balanced and positive conceptualization of ageing-one that emphasizes not only preventing frailty, but actively maintaining robustness as a core scientific and clinical objective.

## Introduction

Frailty is a common geriatric syndrome in older adults, characterized by diminished strength, endurance and physiologic function, which increases vulnerability to adverse health outcomes (1–3). Many studies examined frailty transitions over time (4–12). Although the negative consequences of frailty are well known clinical interventions are often too late for effective management (13–16). Therefore, it is important to identify factors that can help maintain the robustness of older adults.

Robustness represents the opposite pole of frailty and refers to the capacity to maintain physiological reserve, adaptability, and functional integrity despite age-related challenges(17, 18). In the context of frailty research, robustness has traditionally been defined as being “non-frail” (19). However, recent conceptual frameworks propose that robustness should be viewed as an active and multidimensional state encompassing physical, cognitive, psychological, and social domains (13, 20). This broader view positions robustness not merely as the absence of frailty but as a positive expression of intrinsic capacity and adaptive potential (21, 22).

Various modifiable behaviors and lifestyle characteristics have been identified to protect against frailty and maintain robustness. For example, physical activity is consistently associated with lower incidence of frailty in a dose dependent manner such that higher levels of activity are associated with lower odds of developing frailty (23–25). Social participation and social support have also repeatedly been associated with slower frailty progression and lower disability and mortality risk (10, 26), whereas social isolation and loneliness show mixed but largely negative consequences for frailty progression (26, 27). Effects of diet on robustness have been mixed (24, 28) as has the effect of varying degrees of alcohol consumption (29), but there is consistent evidence showing that smoking cessation can promote robustness (27, 30). These examples highlight that while some lifestyle domains show mixed or context-dependent effects on robustness, a core set of modifiable behaviours consistently emerge as protective factors that help older adults remain robust. However, large, longitudinal, multi-domain analyses which focus on sustained robustness are lacking (31–33).

The added value of the present study lies in its design rather than in proposing a new biological construct. Using eight years of longitudinal SHARE data from 27 European countries, we examine sustained robustness among older adults who were robust at baseline and assess a broad set of demographic, socioeconomic, health, physical, social, and cognitive factors within a single analytic framework. In addition, by stratifying the sample into younger-old and older-old adults, the study allows comparison of whether the correlates of maintained robustness differ across stages of later life.

Most studies focus on predicting frailty, worsening states, and adverse effects, whereas the determinants of sustained robustness are less studied (2, 3, 34) and warranted (2, 3, 34) Building on this foundation, the present study directly responds to these gaps by adopting a multidimensional and longitudinal approach that explicitly examines sustained robustness, rather than the development of frailty. Using data from the Survey of Health, Ageing, and Retirement in Europe (SHARE) - a comprehensive eight-year multinational dataset (35), we examined how demographic, health, and lifestyle characteristics interact over time to preserve robustness. Unlike previous longitudinal studies, our analysis identifies potential protective pathways and modifiable factors associated with sustained robustness, representing a conceptual and empirical shift from describing decline to understanding how robustness may be maintained in later life.

This study operationalizes robustness as a dynamic and measurable construct, integrating behavioral, psychosocial, and health-related domains within a unified framework. By emphasizing the determinants of sustained robustness rather than the predictors of frailty, it introduces a conceptual and methodological shift in ageing research. This approach moves beyond risk reduction toward the proactive maintenance of function and independence, positioning robustness as an active and multidimensional outcome of protective factors. Together, these perspectives highlight the importance of modifiable behavioral and social pathways for promoting healthy ageing and provide an empirical foundation for targeted, preventive interventions.

Although the present study does not examine biological mechanisms directly, it contributes to the gerontology literature by showing how readily measured clinical, behavioral, and socioeconomic factors are associated with sustained robustness over time. This population-level perspective is important because it identifies observable markers and potentially modifiable domains that may help distinguish older adults who maintain robustness from those who transition toward pre-frailty or frailty.

## Methods

### Study Design and Data Source

This study is based on a longitudinal analysis using data from Waves 4–8 (2011–2020) of SHARE (35–43). SHARE is a multinational, population-based panel study designed to provide insights into aging, health, and socioeconomic factors across Europe. Since its inception in 2004, SHARE has collected data from over 140,000 participants across 27 countries, making it one of the most comprehensive datasets for aging research.

## Study Population

Our study sample included adults who were classified as robust (non-frail) at Wave 4 of SHARE, which was used as the baseline reference point for tracking robustness Participants were included in the analysis only if they had frailty phenotype data at Wave 4 and at least one other wave, allowing for longitudinal assessment of robustness-frailty transitions. Participants were aged 65–84, stratified into two subgroups: 65–74 years (younger-old) and 75–84 years (older-old). The age stratification is based on previous research on ageing trajectories (e.g. (44), (45), and (46). Typically, three groups are defined (65–74, 75–84, and 85+); here we focus on the younger two groups.

## Frailty Phenotype Assessment

Frailty status was determined using a modified frailty phenotype model based on the five criteria originally proposed by (19) and adapted for SHARE (47). In this model, robustness represents the opposite end of the frailty spectrum individuals classified as “robust” met none of the following frailty criteria based on the original phenotype proposed by (19), indicating preserved strength, energy, and functional reserve.

1. Grip strength of both hands assessed using a handheld dynamometer (Smedley, TTM, Tokyo), with the maximum value across trials retained (Maxgrip, SHARE protocol). If grip strength fell below the 20th percentile (very weak), adjusted for sex and BMI, as recommended by (47), participants received a point, indicating frailty.
2. Walking speed measured using a 2.5-meter walking test, with timing starting when the first foot crossed the start line. Participants in the lowest 20th percentile, adjusted for sex and height (47), received a point, indicating frailty.
3. Unintentional weight loss, defined as self-reported weight loss of 10 lbs (4.5 kg) or more in the past year indicated frailty (1 point). If weight loss data were unavailable, responses to appetite-related survey questions were used as proxies, as previously suggested (47).
4. Exhaustion, assessed using two items from the Center for Epidemiological Studies Depression (CES-D) scale: “I felt everything I did was an effort” and “I could not get going.” Participants reporting these symptoms for at least 3–4 days per week received a point, indicating frailty.
5. Physical activity, based on self-report engagement in low or moderate-intensity activities such as walking, gardening, or household chores. Participants engaging in such activities less than once a week received a point in alignment with SHARE-based studies.

Points of these five criteria were summed up and classified: 0 points - Robust (non-frail), 1–2 points -Pre-frail, 3 or more points - frail.

## Covariates and Control Variables

To comprehensively examine factors predicting sustained robustness, demographic, socioeconomic, health, and lifestyle variables were included as covariates, selected for their relevance to robustness research and availability within the SHARE dataset. All variables included in the analysis were selected based on the factors identified in the literature as key determinants of frailty and robustness, as discussed in the Introduction. Specifically, demographic, socioeconomic, health, and lifestyle characteristics-such as age, sex, education, financial status, chronic diseases, self-rated health, physical activity, and social participation have consistently been linked to frailty progression or robustness maintenance in previous studies (2, 3, 10, 24, 26–30, 34, 48). Accordingly, these variables were extracted from the SHARE dataset for the present analysis.

## Demographic and Socioeconomic Factors

Key demographic variables included sex and socioeconomic status, assessed using education level (years of formal schooling) and financial situation, measured through self-reported ability to “make ends meet” on a scale from financial difficulty to financial security. Marital status (married or single/widowed/divorced) was also examined. In addition, living arrangement was captured using a binary variable (“single”), defined as living alone regardless of formal marital status. Accordingly, individuals classified as “single” could be never married, divorced, widowed, or married, provided they lived alone.

### Health-Related Variables

Health status was measured by the number of diagnosed chronic conditions reported, an indicator of multimorbidity widely used in ageing research (2, 49). Perceived health status was assessed using a self-rated health scale ranging from 1 (excellent) to 5 (poor), a validated and widely recognized predictor of morbidity, mortality, and overall functional decline (50–52). Both indicators were included given their consistent associations with frailty progression and robustness maintenance in previous longitudinal studies.

## Physical Activity and Functional Status

Physical activity was categorized into vigorous activity (e.g., participation in sports or heavy physical labor) and moderate activity (e.g., walking, gardening, or household chores). The frequency of each type of activity was rated from 1 = Hardly ever or never, 2 = One to three times a month, 3 = Once a week, and 4 = More than once a week. Higher ratings indicate greater engagement in physical activity, with “4” representing regularly active individuals and “1” representing those who rarely or never engage in such activities.

Physical activity has been repeatedly shown to reduce frailty risk and promote robustness (24, 25, 48), therefore it was included as a core behavioral variable to capture its protective influence on maintaining robustness and functional independence.

## Social and Cognitive Engagement

Social participation was evaluated through engagement in volunteer work, attendance at educational or training courses, and participation in community-related or recreational activities such as sports clubs, religious, or political organizations. Cognitive engagement was assessed through participation in activities such as reading, solving puzzles (e.g., crosswords, Sudoku), and playing strategy-based games (e.g., chess, card games). Religious engagement (e.g., prayer frequency) was also considered, given its potential association with social support and psychological well-being.

These indicators were selected based on evidence linking social participation and cognitive stimulation to reduced frailty progression and improved functional outcomes in later life (10, 26, 53–55). Such activities have been shown to contribute to the maintenance of cognitive and psychosocial functioning, thereby supporting sustained robustness across ageing populations.

### Statistical Analysis

We used a series of logistic regression models to identify baseline sociodemographic, health, behavioral, and engagement factors associated with remaining robust over time. Specifically, we examined the likelihood of transitioning from robust to pre-frailty or frailty (binary variable) across four follow-up intervals (every two years starting from 2011). Models were stratified by age groups (65–74 and 75–84) to enable age-sensitive interpretation. Odds ratios (OR) greater than 1 indicate higher odds of remaining robust, while values below 1 indicate reduced odds. For health-related variables, higher values in both chronic conditions (0–9) and self-perceived health (1 = excellent to 5 = poor) represent worse health, so ORs less than 1 reflect the expected negative association with robustness.

## Results

Our sample included 7,865 adults; younger-old adults aged 65–74 (n = 5,811, 73.9% of the cohort; 49.9% female), and older-old adults, aged 75–84 (n = 2,054, 26.1% of the cohort; 53.4% women). Groups are characterized in Table 1. There were significant differences in health parameters; the younger-old group was characterized by better health. Both groups participated in social activities, though the younger-old group was significantly more active. The older-old group participated in significantly more physical activity and had lower BMIs, on average, than the younger-old group (Table 1).

**Figure 1:**
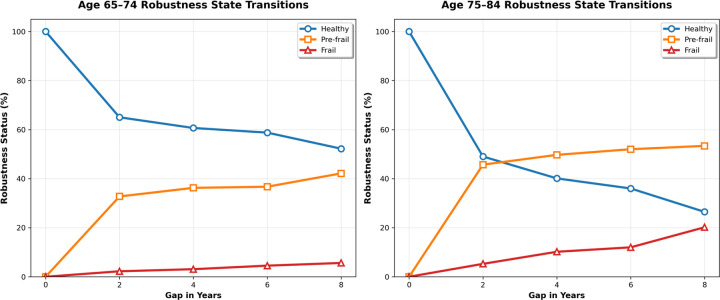
Transition from robustness to frailty across the study period, stratified by age group.

We followed each participant’s robustness over time and assessed changes in the distribution of health states across subsequent waves (see Table 2). At baseline, all participants were classified as robust. Over the eight-year follow-up, the younger-old group declined from 100% robustness at baseline to 52.2% remaining robust, 42.2% transitioning to pre-frailty, and 5.6% to frailty by Wave 8. The older-old group showed a steeper decline, with only 26.5% remaining robust, 53.4% classified as pre-frail, and 20.2% as frail at the end of the study period. These patterns indicate a progressive loss of robustness with age, with faster transitions toward pre-frailty and frailty among the older-old cohort. As illustrated in Fig. 1, these longitudinal transitions demonstrate a clear age-stratified shift from robustness toward pre-frailty and frailty across successive SHARE waves.

As shown in Table 3, logistic regression analyses identified baseline sociodemographic, health, behavioral, and engagement factors associated with sustained robustness across waves and age groups.

Several variables were associated with robustness across cohorts. Engagement in social, cognitive, and physical activities emerged as partially protective, though patterns varied by age group and wave. For example, participation in sporting or social clubs was strongly associated with remaining robust among younger-old adults (e.g., OR = 1.259, p < 0.001 in Wave 4–5). Among older-old adults, participation in educational courses or trainings (OR = 2.288, p = 0.012 in Wave 4–7) and involvement in community organizations (OR = 1.67, p = 0.030 in Wave 4–6) were also linked to sustained robustness. Cognitive activities, such as reading, were associated with greater robustness across the study period for younger-old adults (OR = 1.34, p = 0.008), though interestingly, the association was inverse among older-old adults (OR = 0.551, p = 0.008).

Physical activity contributed in complementary ways. Moderate activities such as walking or gardening consistently predicted higher robustness (e.g., OR = 1.231, p = 0.017 in Wave 4–6), while vigorous activity was beneficial primarily during earlier follow-ups and among younger-old (OR = 1.052, p = 0.025 at Wave 4–5) and older-old adults (OR = 1.116, p = 0.003 at Wave 4–5). However, the protective effect of physical activity appeared to weaken slightly over time, particularly for vigorous activity, which lost significance in later waves. In contrast, moderate activity remained consistently associated with robustness, suggesting that sustainable, age-appropriate physical engagement plays a more enduring role in maintaining robustness in later life.

Lifestyle behaviors showed less consistent associations. Smoking and alcohol use did not demonstrate stable relationships with robustness, apart from baseline smoking among older-old adults between Waves 4 and 6, which unexpectedly predicted higher robustness (OR = 1.31, p = 0.018). BMI displayed weak and inconsistent effects, with lower values marginally associated with robustness in shorter follow-ups (e.g., OR = 0.967, p < 0.001 in Wave 4–5 for older-old; OR = 0.975, p = 0.044 in Wave 4–7 for younger-old) but no significant impact by Wave 4–8.

Self-rated health emerged as the strongest determinant of sustained robustness across all follow-up waves. Participants who perceived their health more favorably (“1” on the scale, indicating better health) had significantly higher odds of remaining robust regardless of age or wave (e.g., younger-old: OR = 0.719, p < 0.001; older-old: OR = 0.714, p < 0.001 in Wave 4–5). In contrast, each additional chronic condition reported at baseline reduced the likelihood of maintaining robustness across both age groups (e.g., younger-old: OR = 0.890, p < 0.001; older-old: OR = 0.905, p = 0.015 in Wave 4–6).

Finally, financial distress emerged as an important but distinct predictor of robustness. Participants reporting lower financial distress (greater financial security) had higher odds of remaining robust, particularly among younger-old adults (e.g., OR = 1.109, p = 0.018 in Wave 4–8). Although this factor is less modifiable than behavioral or health-related variables, it underscores that both objective indicators (such as multimorbidity) and subjective perceptions (self-rated health) jointly influence the likelihood of maintaining robustness.

## Discussion

Using the SHARE data, robustness declined steadily over the eight-year follow-up, with 52% of younger-old adults (65–74) and 26% of older-old adults (75–84) remaining robust by the final wave. Better self-rated health, fewer chronic conditions, greater participation in physical, cognitive, and social activities, and lower financial distress were all associated with a higher likelihood of maintaining robustness over time. Moderate physical activity and social engagement showed the most consistent protective effects, while traditional clinical risk factors such as BMI, smoking, and alcohol consumption were not significantly related to sustained robustness. These findings and the multidimensional nature of robustness are discussed below.

Robustness, increasingly recognized as the counterpart to frailty, reflects preserved physical, cognitive, and psychosocial function in later life (17–19). Drawing on eight years of longitudinal follow-up data from the SHARE study (Waves 4–8), this research examined trajectories of robustness and frailty among adults aged 65–84, divided into two age classes, across four observation intervals. By following individuals who remained robust over time, we were able to identify the factors most strongly associated with maintaining robustness in later life. This research addresses a key gap in the literature, where most studies have focused primarily on the onset and progression of frailty. It advances the field by identifying modifiable protective factors, such as perceived health, financial security, and social or cognitive engagement, that help sustain robustness over time (3, 56). The main contribution of this study is empirical and comparative. Whereas much of the frailty literature emphasizes deterioration, transition to frailty, or adverse outcomes, the present analysis begins with older adults who were robust at baseline and asks which factors are associated with preserving that state over an extended follow-up. By doing so across 27 countries and in two age strata, the study provides evidence that sustained robustness is shaped by multiple domains simultaneously and that the relative importance of these domains may differ between younger-old and older-old adults.

The baseline cohort data collected at Wave 4 of older adults who are robust reflects a relatively well-educated and active sample of adults aged 65–74 (younger-old) and 75–84 (older-old). Previous longitudinal studies on ageing and frailty have typically focused on narrower or more specific age groups-such as younger participants aged 60–75 years (e.g.,(57)) or older cohorts aged 70–90 years (e.g.,(7, 8, 58)) limiting their ability to generalize findings across different stages of ageing. Our cohort, by contrast, bridges this gap by simultaneously examining younger-old and older-old adults within the same long-term framework.

To contextualize these findings within the broader ageing literature, several large-scale longitudinal studies have examined frailty transitions and health trajectories among older adults, providing comparative insights into robustness maintenance across different populations. In the Newcastle 85 + Study (8), which followed 845 individuals aged 85 to 90, only 28% remained robust after five years-highlighting frailty progression but providing little insight into the protective mechanisms sustaining robustness. Similarly, (7), analyzing 25,446 Europeans aged 65 + in SHARE, demonstrated that frailty indices increased sharply beyond age 75, particularly among those with lower education levels, confirming a European “tipping point” for health deterioration. However, this study focused primarily on frailty progression rather than robustness maintenance. Chen (59), using data from 37,264 participants in the Chinese Longitudinal Healthy Longevity Survey, found that higher childhood and adult socioeconomic status predicted 4–6 additional years of healthy longevity, yet the study did not distinguish between robust and pre-frail trajectories. Similarly, (11), analyzing over 2.1 million English adults aged 50+, showed that frailty prevalence rose from 26.5% to 38.9% between 2006 and 2017, but the study’s design was cross-sectional in nature, limiting causal inference regarding long-term robustness maintenance.

Despite age-related health declines, participants in our study demonstrated substantial engagement in physical and social activities. This pattern is consistent with findings from the DO-HEALTH trial, where 23% of 1,889 participants aged ≥ 70 was classified as “healthy agers” at baseline and 65% maintained or improved their status after four years (60). However, DO-HEALTH was a four-year interventional trial, limiting its ability to assess naturalistic long-term ageing trajectories. Likewise, (5) reported that 61.8% of robust adults in SHARE remained robust after two years and that loneliness predicted deterioration-important insights, but within a short timeframe. By leveraging eight years of continuous SHARE data across 27 countries, our study provides one of the most extended and comprehensive assessments of robustness maintenance, offering a proactive perspective that complements these shorter-term or frailty-focused investigations.

While (4) reported that 69.1% of adults > 50 remained robust over eight years in the Taiwanese TLSA cohort, their sample was considerably young (mean 63.4 years). Similarly, (9) observed 69.2% robustness retention over 5.2 years (mean age 75.9), but their shorter follow-up and regional Spanish cohort likely account for higher stability rates. Our SHARE-based study included older adults aged 65–84 and applied a multidimensional framework integrating physical, cognitive, and psychosocial factors to explain sustained robustness. Our findings also reveal engagement in cognitive activities (e.g., reading, puzzles, games) and social participation (e.g., clubs, volunteering) were linked to higher odds of robustness, especially in earlier waves and among younger-old adults. These associations support theories of cognitive reserve and social buffering and align with previous findings showing that mental activity promotes functional health (2, 53–55, 61, 62). However, their effects declined with age, perhaps suggesting the importance of early and sustained engagement.

Physical activity also emerged as an important determinant of sustained robustness. Consistent with prior evidence, moderate activity (e.g., walking, gardening) was strongly associated with remaining robust across waves, while vigorous activity showed benefits primarily among younger-old adults (23, 63–66). These findings support the notion that even moderate, routine physical activity helps preserve functional reserve and delays frailty onset, particularly when from early old age. The differential impact of vigorous activity suggests that age-appropriate and sustainable forms of exercise may be more strongly associated with maintaining robustness in ageing populations.

In contrast, traditional clinical risk factors - such as smoking and BMI - showed only limited or inconsistent associations with remaining robust across waves. Smoking was found to be protective in the older-old participants, seemingly in contrast to common sense, as well as the body of literature, which links smoking and frailty (67, 68). This relationship was significant only when looking at the Wave 4–6 transition; on the one hand it could represent increased social encounters (social smoking), but more likely, it is an anomaly, as the association was not observed in any other models. We did not observe an effect of alcohol consumption (2 + drinks almost every day) on robustness in any waves; previous research found 1–7 alcoholic drinks per week to be associated with reduced frailty (69), suggesting that a more granular examination of alcohol consumption may be required.

Taken together, these findings suggest that the maintenance of robustness in later life is driven by the interplay of physical, cognitive, and social pathways rather than by any single behavioral domain. Moderate physical activity may enhance cardiovascular and neuromuscular efficiency, supporting the energy and mobility required for social participation and cognitive engagement. Similarly, participation in social clubs and community activities can reinforce motivation for sustained physical activity and provide emotional support that buffers stress and inflammation. Cognitive engagement, in turn, contributes to self-regulation, executive functioning, and planning-facilitating adherence to active and socially connected lifestyles. This synergy between physical, cognitive, and social mechanisms forms a multidomain protective model of ageing, in which regular movement, meaningful interaction, and continuous mental stimulation jointly sustain functional independence and robustness.

While clinical risk factors were not associated with robustness here, self-rated health consistently emerged as the strongest protective factor for maintaining robustness across waves and age groups. Older adults who perceived their health more favorably at baseline were more likely to remain robust throughout follow-ups, echoing previous work on the predictive value of subjective health assessments on ageing trajectories. Beyond its subjective nature, self-rated health may function as an integrative signal of underlying biological ageing processes. Previous research suggests that this measure captures multisystem dysregulation, cumulative inflammatory burden, and functional reserve that are not fully reflected by diagnosed conditions alone. From a geroscience standpoint, self-rated health may therefore approximate system-level resilience and adaptive capacity, offering a pragmatic population-level indicator of ageing trajectories. Its strong and consistent association with sustained robustness in the present study reinforces its value as a low-cost yet theoretically meaningful marker of long-term resilience in older adults. A significant, negative correlation between frailty and subjective life expectancy was reported in a study of > 1000 older adults (> 50 yrs) in Sub-Saharan Africa (70). Significant correlations between self-rated health and mortality (52) were also found among older adults, 70-year-olds (n = 1008) from Finland. The capacity of this single, easily attained health rating to predict long-term robustness underscores its validity as a pragmatic indicator of resilience in ageing populations. Unlike clinical biomarkers, determining self-rated health requires minimal resources, yet it reliably identifies individuals at elevated risk of maintaining robustness. Incorporating this question into routine primary care, particularly for adults over 75, could help flag those less likely to remain robust and guide earlier monitoring and intervention (50, 51, 71).

Alongside subjective health, economic security represents another critical pillar of sustained robustness in older age. Financial distress was associated with increased frailty risk in nearly all models, particularly among younger-old adults (ages 65–74). Economic insecurity has been linked to stress, reduced access to care, and increased frailty in a number of cohorts studied longitudinally (72, 73) and also in a study on previous waves of the SHARE cohort (Waves 1–5,(74)).

Overall, these findings underscore the multidimensional nature of robustness and point to several practical implications for promoting healthy ageing. Beyond individual behaviors, targeted community-based programs that encourage regular physical and social activity could play a key role in maintaining robustness at the population level. Likewise, financial security interventions such as pension stability, access to affordable healthcare, and social assistance may reduce stress and health vulnerability, thereby reinforcing long-term wellbeing. In clinical settings, the integration of primary-care screening tools that include brief self-rated health assessments and questions on social participation could help identify individuals at risk of decline earlier and enable preventive strategies. Although several SHARE-based studies have examined frailty trajectories, inequalities, and transitions, fewer analyses have focused specifically on the maintenance of robustness among individuals who were robust at baseline. The present study contributes to this literature by combining four elements within a single framework: extended follow-up, broad cross-national coverage, multidomain predictors, and age-stratified analyses. Its uniqueness therefore lies less in identifying new mechanisms than in bringing these elements together to characterize sustained robustness as a measurable population-health outcome in later life.

### Limitations

Frailty and robustness were classified according to slightly stricter operational criteria (47) based on the data collected in SHARE, thereby reducing the likelihood of misclassification and strengthening the robustness of observed associations.

The reliance on some self-reported variables introduces potential recall and reporting bias, though valid and reliable tools were used. In addition, although the study spans multiple waves, its longitudinal structure is based on repeated cross-sectional comparisons, limiting causal inference. Additional variables, such as independence in IADL, caregiving support, nutritional status, or environmental barriers, may have influenced frailty outcomes, but were not included in this analysis. Despite these limitations, our findings highlight modifiable protective factors that are easily identifiable in clinical and community settings, making them valuable targets for early interventions to sustain robustness. The stratification by age groups provided nuanced insights into age-specific dynamics, and the inclusion of functional, psychosocial, and cognitive variables enriched the multidimensional assessment of frailty.

## Conclusions

This study identifies financial security, physical activity, cognitive engagement, and self-rated health as key determinants of sustained robustness among older adults. By shifting the focus from delaying frailty to maintaining robustness, our findings emphasize a multidomain model of healthy ageing, in which behavioral, psychosocial, and economic factors interact to preserve function and independence. These insights highlight the value of community-based programs that foster active and social lifestyles, policies supporting financial stability in later life, and simple primary-care screening tools that assess health perception and participation.

Strengthening these modifiable domains can help extend the years lived in good health. Although SHARE encompasses 27 countries with diverse welfare and healthcare systems, the consistency of our results across contexts underscores the generalizability and robustness of these findings.

## Supplementary Material

Supplementary Files

This is a list of supplementary files associated with this preprint. Click to download.

- SuppTable1.xlsx
- Graphicalabstract.png

## Figures and Tables

**Table 1:** Descriptive statistics of the whole population and differences between groups as assessed at Wave 4 of SHARE

	Whole Population N = 7,865	Younger-old Age 65–74 N = 5,811	Older-old Age 75–84 N = 2,054	Differences between groups	
				t-statistic^1^; χ^2^-statistic^2^	p-value
Age	71.33 ± 4.92	68.90 ± 2.80	78.20 ± 2.58	−130^1^	**p = 0.001**
Sex (female)	3,995 (50.79%)	2,898 (49.87%)	1097 (53.41%)	7.60^2^	**p = 0.006**
Education (years)	10.84 ± 4.35	11.09 ± 4.30	10.12 ± 4.40	8.70^1^	**p = 0.001**
Financial distress (1–4)	3.08 ± 0.86	3.09 ± 0.86	3.05 ± 0.86	1.88^1^	p = 0.06
Chronic disease (number)	1.55 ± 1.31	1.47 ± 1.27	1.81 ± 1.40	−10.25^1^	**p = 0.001**
Self-perceived health status (1–5)	2.84 ± 0.94	2.78 ± 0.95	3.00 ± 0.91	−8.82^1^	**p = 0.001**
Performed voluntary or charity work	1886 (23.99%)	1494 (25.72%)	392 (19.10%)	36.43^2^	**p = 0.001**
Attended an educational or training course	833 (10.60%)	703 (12.10%)	130 (6.34%)	53.26^2^	**p = 0.001**
Belong to a sport, social or other club	2844 (36.18%)	2195 (37.79%)	649 (31.63%)	24.96^2^	**p = 0.001**
Participate in religious activities (church, synagogue, mosque, etc.)	1235 (15.71%)	846 (14.57%)	389 (18.96%)	22.08^2^	**p = 0.001**
Belong to a political or community-related organization	537 (6.83%)	425 (7.32%)	112 (5.46%)	8.24^2^	**p = 0.004**
Read books, magazines or newspapers	6601 (83.98%)	4909 (84.52%)	1692 (82.46%)	4.81^2^	**p = 0.028**
Did word or number games such as crossword puzzles or Sudoku	3993 (50.80%)	3031 (52.19%)	962 (46.88%)	17.08^2^	**p = 0.001**
Played cards or board games (chess)	2655 (33.78%)	2045 (35.21%)	610 (29.73%)	20.38^2^	**p = 0.000**
Attend social activities (0–7)	2.46 ± 1.41	2.54 ± 1.43	2.21 ± 1.34	9.18^1^	**p = 0.001**
Vigorous physical activity	2.35 ± 1.30	2.27 ± 1.28	2.60 ± 1.31	−9.83^1^	**p = 0.001**
Moderate physical activity	1.13 ± 0.34	1.13 ± 0.33	1.14 ± 0.35	−2.16^1^	**p = 0.031**
Daily smoking (yes)	5,419 (68.90%)	4067 (69.99%)	1352 (65.82%)	12.29^2^	**p = 0.001**
Alcohol consumption (> 2 glasses almost every day)	1,378 (17.52%)	1,140 (19.62%)	238 (11.59%)	67.73^2^	**p = 0.001**
Marital status (single)	2,202 (28%)	1,399 (24.08%)	803 (39.09%)	169.82^2^	**p = 0.001**
BMI	26.56 ± 4.01	26.65 ± 4.06	26.29 ± 3.82	3.47^1^	**p = 0.0005**

Significant differences between the two age groups (65–74 vs. 75–84) are in bold. Values are means ± SD. Age is continuous (years). Sex = male/female. Education ranges 0–25 years. Financial distress coded 1 = with great difficulty to 4 = easily, where higher values indicate better financial situation. Chronic diseases: range 0–9. Self-perceived health: coded 1 = excellent to 5 = poor. Activities are dichotomous (0 = no, 1 = yes) for volunteering, courses, clubs, religious and political organizations, reading, puzzles, and games; “Activities” (0–7) is the summed count. Vigorous and moderate physical activity coded 1–4 (1 = hardly ever or never, 2 = one to three times a month, 3 = once a week, 4 = more than once a week). Daily smoking and alcohol use (> 2 glasses almost every day) coded 0 = no, 1 = yes. Marital status = married coded 0, single/divorced/widowed coded 1. BMI is continuous (kg/m^2^).

Variables analyzed using independent-samples t-tests^1^; categorical variables analyzed using chi-square tests^2^.

**Table 2:** The percentage of participants who remained robust or transitioned to pre-frail and frail in Waves 5, 6, 7, 8 for the whole SHARE study population and for each age-group. Baseline is Wave 4, in which all participants were robust.

	Age 65–74			Age 75–84		
	Robust	Pre-Frail	Frail	Robust	Pre-Frail	Frail
Wave 4–5	(65.02%)	(32.74%)	(2.24%)	(49.03%)	(45.71%)	(5.26%)
Wave 4–6	(60.66%)	(36.25%)	(3.09%)	(40.11%)	(49.70%)	(10.19%)
Wave 4–7	(58.77%)	(36.71%)	(4.53%)	(36.00%)	(52.00%)	(12.00%)
Wave 4–8	(52.22%)	(42.15%)	(5.63%)	(26.46%)	(53.36%)	(20.18)

**Table 3:** Logistic regression to identify factors associated with robustness over time, stratified by age group and Wave. Odds ratio ± SD are reported, and significant ratios (p < 0.05) are denoted by grey shading. A full table of model statistics (including z-scores and exact p-values) can be found in Supp Table 1.

	WAVE 4–5		WAVE 4–6		WAVE 4–7		WAVE 4–8	
	Age 65–74	Age 75–84	Age 65–74	Age 75–84	Age 65–74	Age 75–84	Age 65–74	Age 75–84
	N = 5,753	N = 2,015	N = 5,011	N = 1,666	N = 2,074	N = 646	N = 3,264	N = 906
**Age**	0.951 ± 0.009	0.950 ± 0.017	0.934 ± 0.010	0.928 ± 0.019	0.924 ± 0.0155	0.960 ± 0.034	0.936 ± 0.012	0.876 ± 0.029
**Sex** (Female: 0, Male: 1)	1.232 ± 0.078	1.200 ± 0.127	1.237 ± 0.082	1.140 ± 0.137	1.171 ± 0.118	1.300 ± 0.256	1.300 ± 0.105	0.855 ± 0.158
**Education**	1.007 ± 0.007	1.006 ± 0.011	1.008 ± 0.007	1.009 ± 0.012	0.996 ± 0.012	0.994 ± 0.020	1.000 ± 0.008	1.011 ± 0.018
**Single** (No: 0, Yes: 1)	1.041 ± 0.071	0.922 ± 0.096	0.957 ± 0.068	0.936 ± 0.110	1.012 ± 0.117	0.951 ± 0.191	0.959 ± 0.083	1.023 ± 0.181
**Financial distress**	1.167 ± 0.040	1.161 ± 0.065	1.150 ± 0.041	1.102 ± 0.070	1.098 ± 0.062	1.115 ± 0.130	1.109 ± 0.048	1.250 ± 0.126
**Chronic diseases**	0.904 ± 0.021	0.939 ± 0.033	0.890 ± 0.022	0.904 ± 0.037	0.924 ± 0.039	0.849 ± 0.065	0.893 ± 0.028	0.906 ± 0.058
**Self-reported health status**	0.719 ± 0.024	0.714 ± 0.040	0.720 ± 0.026	0.721 ± 0.045	0.728 ± 0.041	0.723 ± 0.074	0.764 ± 0.033	0.717 ± 0.067
**Performed voluntary or charity work**	0.970 ± 0.070	1.092 ± 0.137	1.076 ± 0.085	0.888 ± 0.129	1.028 ± 0.119	1.135 ± 0.243	1.002 ± 0.091	1.032 ± 0.212
**Attended an educational or training course**	1.062 ± 0.101	0.977 ± 0.192	0.974 ± 0.097	1.725 ± 0.376	1.326 ± 0.201	2.288 ± 0.753	1.141 ± 0.131	1.279 ± 0.382
**Belong to a sport, social or other club**	1.259 ± 0.079	1.210 ± 0.126	1.330 ± 0.090	0.893 ± 0.107	1.107 ± 0.114	1.104 ± 0.214	1.107 ± 0.088	1.195 ± 0.211
**Participate in religious activities (church, synagogue, mosque, etc.)**	1.180 ± 0.102	0.965 ± 0.120	1.057 ± 0.091	0.948 ± 0.129	1.198 ± 0.155	1.487 ± 0.308	0.901 ± 0.090	0.943 ± 0.187
**Belong to a political or community-related organization**	1.192 ± 0.145	1.371 ± 0.293	0.989 ± 0.125	1.669 ± 0.395	0.897 ± 0.170	0.898 ± 0.314	0.965 ± 0.138	0.900 ± 0.304
**Read books, magazines or newspapers**	1.001 ± 0.084	1.126 ± 0.149	1.060 ± 0.090	1.553 ± 0.235	1.396 ± 0.182	1.002 ± 0.240	1.340 ± 0.146	0.551 ± 0.124
**Did word or number games such as crossword puzzles or Sudoku**	1.112 ± 0.069	1.177 ± 0.118	1.143 ± 0.075	1.165 ± 0.133	1.040 ± 0.109	1.316 ± 0.253	1.067 ± 0.085	1.352 ± 0.231
**Played cards or board games (chess)**	1.065 ± 0.067	0.858 ± 0.091	1.067 ± 0.071	1.039 ± 0.125	0.882 ± 0.090	1.076 ± 0.212	1.033 ± 0.082	1.020 ± 0.184
**Vigorous physical activity**	1.052 ± 0.024	1.116 ± 0.040	1.042 ± 0.025	1.049 ± 0.043	1.113 ± 0.042	1.095 ± 0.738	1.095 ± 0.032	0.982 ± 0.060
**Moderate physical activity**	1.454 ± 0.121	1.245 ± 0.165	1.231 ± 0.107	1.192 ± 0.188	1.413 ± 0.197	1.274 ± 0.367	1.136 ± 0.124	1.825 ± 0.501
**Smoking**	1.001 ± 0.064	0.991 ± 0.102	0.952 ± 0.063	1.313 ± 0.151	0.427 ± 0.347	N/A	0.957 ± 0.079	1.035 ± 0.182
**Drinking**	0.991 ± 0.074	0.811 ± 0.119	0.922 ± 0.073	0.891 ± 0.149	0.806 ± 0.096	0.883 ± 0.237	0.960 ± 0.093	0.929 ± 0.237
**BMI**	0.994 ± 0.007	0.967 ± 0.012	0.993 ± 0.007	1.002 ± 0.014	0.975 ± 0.012	0.983 ± 0.245	0.982 ± 0.009	0.996 ± 0.021

## Data Availability

The datasets analysed during the current study are available from the SHARE Research Data Center (https://www.share-project.org) and can be accessed by registered researchers upon application in accordance with SHARE data access procedures.
